# Embryonic heat conditioning increases lipolytic gene expression in broiler chicks at day 4 post-hatch

**DOI:** 10.3389/fphys.2024.1445569

**Published:** 2024-09-02

**Authors:** Usman Sulaiman, Reagan S. Vaughan, Paul Siegel, Dongmin Liu, Elizabeth Ruth Gilbert, Mark Andrew Cline

**Affiliations:** ^1^ School of Animal Sciences, Virginia Polytechnic Institute and State University, Blacksburg, VA, United States; ^2^ Department of Human Nutrition, Foods and Exercise, Virginia Polytechnic Institute and State University, Blacksburg, VA, United States; ^3^ School of Neuroscience, Virginia Polytechnic Institute and State University, Blacksburg, VA, United States

**Keywords:** adipose tissue, embryonic heat conditioning, DNA methylation, heat stress, broiler

## Abstract

**Introduction:**

Exposure to elevated temperatures during incubation is known to induce epigenetic changes that are associated with immunological and stress-response differences at a later age. Reports on its effects on the adipose tissue are still scarce. In this experiment, we investigated the effect of embryonic heat conditioning (EHC) on growth, adipose tissue mRNA and global DNA methylation in broiler chicks at day 4 post-hatch.

**Methods:**

Fertile eggs were divided into two groups: control and EHC. Eggs in the control group were incubated at 37.8°C and 80% relative humidity from day 0 to day 18.5 (E0 to E18.5). The EHC eggs were subjected to an intermittent increase in temperature to 39.5°C and 80% relative humidity from E7 to E16 for 12 h (07:30–19:30) per day. On day 4 post-hatch, control and EHC chicks were subjected to 36°C using three time points: 0 (no heat challenge serving as the control), and 2 and 12 h relative to start of the heat challenge. Fifteen chicks were sampled from each group for every timepoint. Body weight was recorded before euthanasia and subcutaneous adipose tissue was collected.

**Results:**

Body weights were similar in control and EHC groups. Diacylglycerol O-acyltransferase 2 (DGAT2) mRNA was lower in the EHC group at time 0 relative to control. Hormone-sensitive lipase (HSL) mRNA was greater in the EHC than control group at the 0 h timepoint. Heat challenge affected adipose tissue DNA methylation, with methylation highest at 12 h into the heat challenge.

**Discussion:**

These findings highlight the dynamic molecular responses of chicks to heat stress during early post-hatch development and suggest that EHC may affect heat stress responses and adipose tissue development through mechanisms involving lipid remodeling and DNA methylation.

## 1 Introduction

Poultry is the main protein source for the majority of the world’s population (Connolly et al., 2022). This can be attributed to its ease of production and quick turnaround time compared to other domestic animals and its acceptance across all religions and cultures. Production is still on the rise yearly and it is expected that it will account for about 41% of the world’s animal protein consumption by 2030 (OECD/FAO, 2021). The quick turnaround time is primarily made possible by selection with short generation intervals and increasing demand for chicken parts as opposed to whole birds in recent decades (Petracci et al., 2015). This selection process made it possible to raise chickens that reach market/table size quickly and produce more robust parts like the breast (Zuidhof et al., 2014). However, this selection is not without unintended consequences. To reach maturity sooner, birds consume more feed and while this is expected, they deposit more adipose tissue than needed. Excess adipose tissue is regarded as a negative trait in poultry production because adipose tissue in excess is not biologically valuable as it diverts energy from the muscles (Claire D’Andre et al., 2013). Asides from that, excess adipose tissue compromises the welfare and health of the birds including their fertility (Bernardi et al., 2021). Solutions have been sought ranging from the supplementation of feeds with different additives, to feed restriction which can become a welfare concern (Fouad and El-Senousey, 2014). Subcutaneous fat is the first depot that is visible during development in broilers, usually by embryonic day 12 (E12). This development is usually triggered by the extraction of fatty acid from the yolk which continues until E19. After E19, a series of events activate lipolysis which converts the stored triglyceride (TG) into energy for the chick to use during the hatching period. Abdominal adipose tissue, which is the primary storage depot of TG in mature chickens develops after hatch, becoming more visible around day 7 post-hatch. (Kim and Voy, 2021).

Heat is widely regarded as a negative phenomenon in commercial poultry production as it hinders growth by reducing feed intake and can lead to death in severe cases. Rising temperature is inevitable due to increased global warming (Yang, 2021). Also, selection for rapid growth over the years gave rise to chickens with impaired thermotolerance (Yahav et al., 2004b). Different techniques have been used to combat this from improved housing to epigenetic programming (Al-Zghoul, 2018). Thermal manipulation of avian species during embryogenesis has been established over the years as a way of altering their stress threshold later in life (Loyau et al., 2016). EHC not only affects the chicks’ stress response post-hatch but also the immunological state and hatchability which has been reported to be higher in heat-conditioned chicks (Halevy et al., 2006; Piestun et al., 2009a; Rajkumar et al., 2015). Since thermal manipulation during embryogenesis has all of these benefits it has been suggested that it could possess other benefits due to cross-tolerance (Farghly et al., 2022). However, research has been scarce in evaluating the effects of thermal epigenetic programming during embryogenesis on adipose tissue development post-hatch. Therefore, the aim of this experiment was to evaluate the molecular mechanisms by which subcutaneous adipose tissue deposition is affected by thermal programming during embryo development. We measured expression of adipose-specific transcription factors and enzymes identified by past studies to have an effect on adipocyte differentiation, lipogenesis, and lipolysis, and assessed global DNA methylation, hypothesizing that changes in embryonic programming and response to heat stress might be epigenetic.

## 2 Materials and methods

### 2.1 Chickens and EHC

All animal protocols were approved by the Institutional Animal Care and Use Committee (IACUC) at Virginia Tech. Fertile chicken eggs of the Cobb-Hubbard cross (*Gallus gallus*) were obtained from a nearby commercial hatchery. Upon arrival at the incubation facility, they were kept at 26.6°C for 12 h after which they were divided into two incubators (Rite Farm Products Pro-1056) labeled control and EHC. The eggs in the control group were incubated at 37.8°C and 80% relative humidity from day 0 to day 18.5 (E0 to E18.5). The EHC group was subjected to an intermittent increase in temperature to 39.5°C and 80% relative humidity from E7 to E16 for 12 h (07:30–19:30) per day. After E18.5, candling took place, and infertile eggs and dead embryos were disposed of and the embryos in good condition were transferred to the hatcher (Rite Farm Products Pro-264) at 36.9°C and 50% relative humidity for 18 h. The temperature was gradually decreased to 35°C until the hatch was collected. Hatched chicks were randomly divided into group cages in preparation for the post-hatch challenge experiment on day 4. The holding room temperature was set at 30°C with 24 h of illumination with *ad libitum* access to food and water. The protocol was based on previous findings in the Cline/Gilbert lab.

### 2.2 Heat challenge

On day 4 post-hatch, both EHC and control groups were subjected to heat challenge at 36°C using three time points: 0 (no heat challenge serving as a control), and 2 and 12 h relative to the start of the challenge. The protocol was developed based on prior research conducted in the Cline/Gilbert lab. Fifteen chicks were randomly sampled from both the control and EHC groups for a total of 30 chicks sampled at each time point. Chicks were individually weighed and euthanized by decapitation. Sex was determined by gonadal inspection and adipose tissue samples were collected as described below.

### 2.3 Tissue collection

Subcutaneous fat was collected from the breast wing axis. This was done by removing the skin to expose the fat pads and sterile forceps and scissors were used to obtain the sample. The workstation and instruments were cleaned using 70% ethanol and sterile wipes between each sample collection to avoid cross-contamination. Adipose tissue samples were rinsed in ice-cold phosphate-buffered saline, blotted on a wipe, and immediately submerged in RNALater (Fisher Healthcare, Houston, TX, United States) for RNA isolation and Lysis Buffer B (Norgen Biotek, Thorold, ON, Canada) for genomic DNA extraction. They were incubated at 4°C overnight and then moved to −20°C until total RNA and DNA extraction.

### 2.4 Total RNA extraction and cDNA synthesis

Adipose tissue samples (200 mg) were homogenized in TRI Reagent (Sigma-Aldrich, St. Louis, MO, United States) with 5 mm stainless steel beads (Qiagen, Valencia, CA, United States) using a Tissue Lyser II (Qiagen). At the step of addition of ethanol (molecular-biology grade; Fisher Healthcare), total RNA was isolated using the Zymo Quick-DNA/RNA Miniprep kit (Zymo Research, Irvine, CA, United States) following the manufacturer’s instructions. The concentration and purity of the isolated total RNA were checked using the Nanophotometer Pearl Spectrophotometer (Implen, Westlake Village, CA, United States) at 260/280/230 nm. The cDNA was synthesized from 200 ng of total RNA with the High Capacity cDNA Reverse Transcription Kit (Applied Biosystems, Carlsbad, CA, United States). Reactions were performed under the following conditions: 25°C for 10 min, 37°C for 120 min, and 85°C for 5 min.

### 2.5 Real-time quantitative PCR (RT-qPCR)

Primers for real-time PCR (Table 1) were designed using Primer Express (Applied Biosystems, Carlsbad, CA, United States). Real-time PCR was carried out in duplicate of 10 μL volume reactions that contained 5 μL Fast SYBR Green Master Mix (Applied Biosystems, Carlsbad, CA, United States), 0.25 μL each of 5 μM forward and reverse primers, and 3 μL of 10-fold diluted cDNA using a 7500 Fast Real-Time PCR System (Applied Biosystems, Carlsbad, CA, United States). The PCR was performed under the following conditions: 95°C for 20s and 40 cycles of 90°C for 3s plus 60°C for 30s. A dissociation step consisting of 95°C for 15s, 60°C for 1 min, 95°C for 15s, and 60°C for 15s was performed at the end of each PCR reaction to ensure amplicon specificity. Real-time PCR data were analyzed using the ΔΔCT method, where.

**TABLE 1:** Primers for real-time PCR.^a^.

Gene	Sequences (forward/reverse)	Accession no.
β-actin	GTC​CAC​CGC​AAA​TGC​TTC​TAA/TGC​GCA​TTT​ATG​GGT​TTT​GTT	NM_205518.2
*C/EBPα*	CGC​GGC​AAA​TCC​AAA​AAG/GGC​GCA​CGC​GGT​ACT​C	NM_001031459.2
*C/EBPβ*	GCC​GCC​CGC​CTT​TAA​A/CCA​AAC​AGT​CCG​CCT​CGT​AA	NM_205253.3
*DGAT2*	TTG​GCT​TTG​CTC​CAT​GCA​T/CCC​ACG​TGT​TCG​AGG​AGA​A	XM_040661932.1
*LPL*	GAC​AGC​TTG​GCA​CAG​TGC​AA/CAC​CCA​TGG​ATC​ACC​ACA​AA	NM_205282.2
*PPARγ*	CAC​TGC​AGG​AAC​AGA​ACA​AAG​AA/TCC​ACA​GAG​CGA​AAC​TGA​CAT​C	NM_001001460.2
*SREBP1*	CAT​CCA​TCA​ACG​ACA​AGA​TCG​T/CTC​AGG​ATC​GCC​GAC​TTG​TT	NM_204126.3
*HSL*	GCG​GTG​CTG​AGG​GAG​TAC/CCC​GAG​ACA​CCT​CCC​ATA​GA	XM_040657096.1
*ATGL*	GCC​TCT​GCG​TAG​GCC​ATG​T/GCA​GCC​GGC​GAA​GGA	NM_001113291.2
*MGLL*	GCG​GAC​GAG​CGT​AGA​CTC​A/GGG​AAT​AGC​CTG​GTT​TGC​AA	NM_001277142.2
*NPY*	CAT​GCA​GGG​CAC​CAT​GAG/CAG​CGA​CAA​GGC​GAA​AGT​C	NM_205473.2

^a^ Abbreviation: C/EBPα, CCAAT/enhance binding protein α; C/EBPβ, CCAAT/enhancer binding protein β; DGAT2, diacylglycerol O-acyltransferase 2; LPL, lipoprotein lipase; PPARγ, peroxisome proliferator-activated receptor γ; SREBP1, sterol regulatory element-binding transcription factor 1; HSL, hormone-sensitive lipase; ATGL, adipose triglyceride lipase; MGLL, monoacylglycerol lipase; NPY, Neuropeptide Y.

ΔCT = CT target gene − CT actin, and ΔΔCT = ΔCT target sample − ΔCT calibrator.

The average of the control chicks was used as the calibrator sample. The fold difference was calculated as 2^^–ΔΔCT^.

### 2.6 Genomic DNA extraction

Adipose tissue samples were homogenized into a fine powder in liquid nitrogen using a mortar and pestle and then transferred into a nuclease-free microcentrifuge tube. Genomic DNA was isolated using the Cells and Tissue DNA Isolation Kit (Norgen Biotek, Thorold, ON, Canada) following the manufacturer’s instructions. The concentration and purity of the total DNA were assessed using a Nanophotometer Pearl spectrophotometer (Implen, Westlake Village, CA, United States) at 260/280/230 nm.

### 2.7 Global DNA methylation quantification

Global DNA methylation was quantified using the MethylFlash™ Global DNA Methylation (5-mC) ELISA Easy Kit (Colorimetric) (Epigentek, Farmingdale, NY, United States) following the manufacturer’s instructions. The amount of input DNA was 100 ng per reaction. The absorbance was measured at 450 nm, and the percentage of DNA methylation (5-methyl cytosine %; 5-mC%) was calculated using the formula:

$$5‐\text{mC }\%=\frac{\left(\text{Sample OD}‐\text{ Negative control OD}\right)÷100\text{ ng DNA input }}{\;\left(\text{Positive control OD}‐\text{ Negative control OD}\right)\times 2÷5\text{ ng Positive control}}$$

### 2.8 Statistical analysis

Body weight (BW) data were analyzed using the Fit Model of JMP Pro 16 (SAS Institute Inc., Cary, NC), and the model included the effect of treatment (EHC vs control), time (0, 2, and 12 h), and the interaction between them.

PCR data were analyzed with the Fit Model using JMP Pro 16 (SAS Institute Inc., Cary, NC). For data at time 0 (baseline), the model included the effect of treatment (control vs EHC). For analyzing effects of the heat challenge, the model included the effect of time (0, 2, and 12 h) within each treatment group (control and EHC). Initial analyses that included the whole model (2-way ANOVA testing for effects of incubation treatment, heat challenge time, and 2-way interaction) did not yield any significant differences; thus, final analyses considered the effects of treatment at baseline, and effects of heat challenge within treatment.

The 5-mC results were further analyzed using the Fit Model in JMP Pro 16 (SAS Institute Inc., Cary, NC). The statistical model for the 5-mC data included the effects of treatment (EHC vs. control), time (0, 2, and 12 h), and the interaction between them.

Tukey’s test was used for all *post hoc* comparisons and differences were assigned at *p* < 0.05.

## 3 Results

### 3.1 Growth performance

**FIGURE 1:**
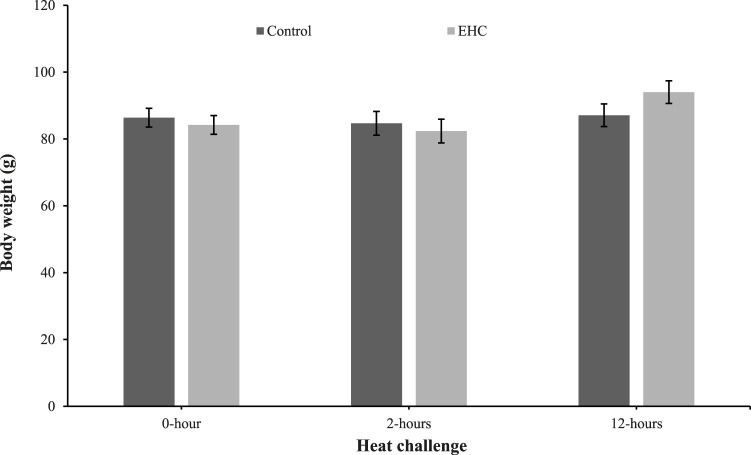
Effect of heat challenge within time on body weight of control and EHC chicks on day 4 post-hatch. Data (n = 15/group) were analyzed using the Fit Model of jMP and presented as means ± SEM. Chicks hatched at 37.8°C (Control) from day 0 to day 18.5 (E0 to E18.5) or intermittent increase in temperature to 39.5°C and 80% relative humidity from E7 to E16 for 12 h - 07:30–19:30 (EHC). Candling at E18.5 and temperature set to 36.9°C and 50% relative humidity until hatch. Heat challenge = 36°C using three time points: 0 (no heat challenge serving as a control), and 2 and 12 h relative to the start of the challenge.

As shown in Figure 1, the body weights of the control and EHC chicks were similar. These results indicate that the EHC and heat challenge did not affect body weight at day 4 post-hatch.

### 3.2 Adipose tissue mRNA

**FIGURE 2:**
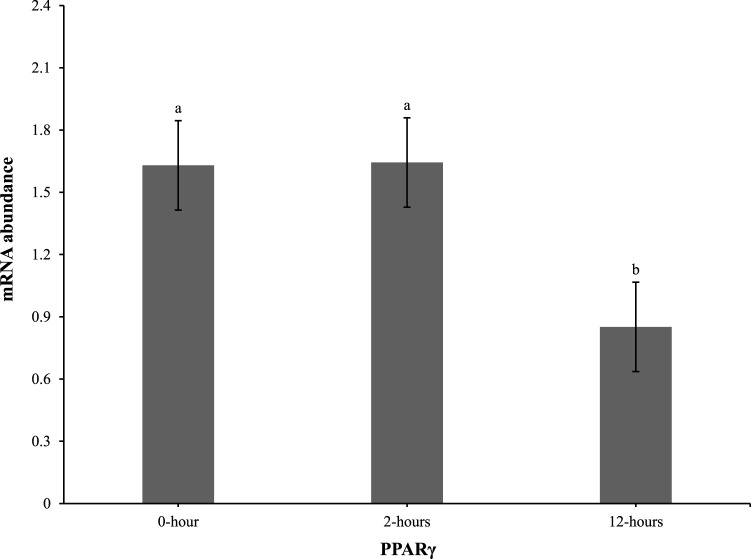
Effect of heat challenge on mRNA abundance of DGAT2 in the subcutaneous adipose tissue of embryonic heat condition (EHC) chicks on day 4 post-hatch. Data (n = 15/timepoint) was analyzed using the Fit Model of jMP and presented as means ± SEM. Different superscripts between timepoints show a difference at *p* < 0.05; Tukey’s test. Chicks hatched at 37.8°C (Control) from day 0 to day 18.5 (E0 to E18.5) or intermittent increase in temperature to 39.5°C and 80% relative humidity from E7 to E16 for 12 h - 07:30–19:30 (EHC). Candling at E18.5 and temperature set to 36.9°C and 50% relative humidity until hatch. Heat challenge = 36°C using three time points: 0 (no heat challenge serving as a control), and 2 and 12 h relative to the start of the challenge.

**FIGURE 3:**
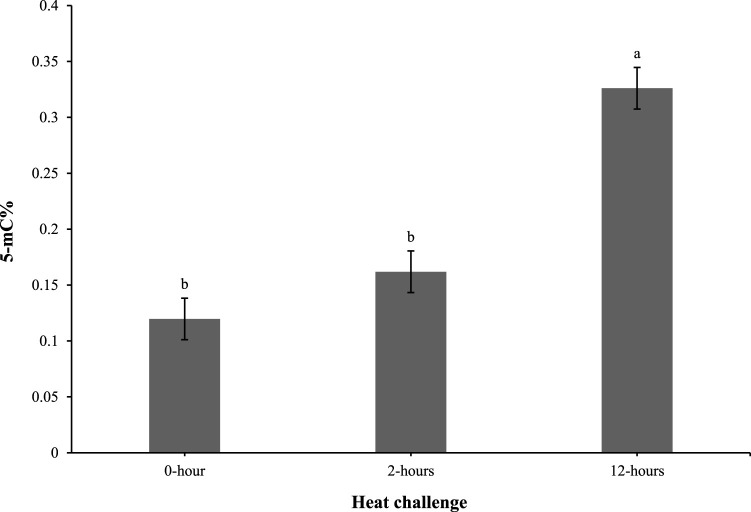
Effect of heat challenge on mRNA abundance of PPARγ in the subcutaneous adipose tissue of embryonic heat condition (EHC) chicks on day 4 post-hatch. Data (n = 15/timepoint) was analyzed using the Fit Model of jMP and presented as means ± SEM. Different superscripts between timepoints show a difference at *p* < 0.05; Tukey’s test. Chicks hatched at 37.8°C (Control) from day 0 to day 18.5 (E0 to E18.5) or intermittent increase in temperature to 39.5°C and 80% relative humidity from E7 to E16 for 12 h - 07:30–19:30 (EHC). Candling at E18.5 and temperature set to 36.9°C and 50% relative humidity until hatch. Heat challenge = 36°C using three time points: 0 (no heat challenge serving as a control), and 2 and 12 h relative to the start of the challenge.

At 0 h post-heat challenge (Table 2), the relative mRNA expression levels of CCAAT/enhance binding protein α (C/EBPα), DGAT2, peroxisome proliferator-activated receptor γ (PPARγ), sterol regulatory element-binding transcription factor 1 (SREBP1), HSL, and neuropeptide Y (NPY) showed significant differences between the control and EHC groups. Additionally, Figures 2, 3 show the expression of DGAT2 and PPARγ respectively across the three timepoints for the EHC chicks. Figure 2 highlights an elevated expression of DGAT2 at 2 h post-heat challenge, which returned to levels similar to those at 0 h by 12 h post-heat challenge. In Figure 3, the expression levels of PPARγ remained constant at the 0 and 2-h time points. However, expression decreased by 12 h post-heat challenge.

**TABLE 2:** Means and standard errors of heat challenge on relative mRNA abundance of adipocyte factors in subcutaneous adipose tissue.

Timepoints^a^	*C/EBPα*	*C/EBPβ*	*DGAT2*	*LPL*	*PPARγ*	*SREBP1*	*HSL*	*ATGL*	*MGLL*	*NPY*
0 h										
Control	1.16 ± 0.15	1.07 ± 0.11	1.44 ± 0.25	1.36 ± 0.24	2.25 ± 0.26	1.32 ± 0.18	0.76 ± 0.19	1.06 ± 0.21	1.09 ± 0.13	1.25 ± 0.15
Treatment	0.61 ± 0.17	0.99 ± 0.11	0.69 ± 0.23	1.21 ± 0.24	1.38 ± 0.24	0.75 ± 0.18	1.32 ± 0.18	1.28 ± 0.21	1.03 ± 0.13	0.75 ± 0.16
*p*-value	**0.0260**	0.6286	**0.0416**	0.6650	**0.0209**	**0.0355**	**0.0442**	0.4510	0.7311	**0.0331**

^a^ Values represent means and standard errors of the means with associated *p*-values for the effect of embryonic heat treatment on adipose tissue depot (n = 15/group).

Abbreviation: C/EBPα, CCAAT/enhance binding protein α; C/EBPβ, CCAAT/enhancer binding protein β; DGAT2, diacylglycerol O-acyltransferase 2; LPL, lipoprotein lipase; PPARγ: peroxisome proliferator-activated receptor γ; SREBP1, sterol regulatory element-binding transcription factor 1; HSL, hormone-sensitive lipase; ATGL, adipose triglyceride lipase; MGLL, monoacylglycerol lipase; NPY, Neuropeptide Y.

### 3.3 Global DNA methylation quantification

**FIGURE 4:**
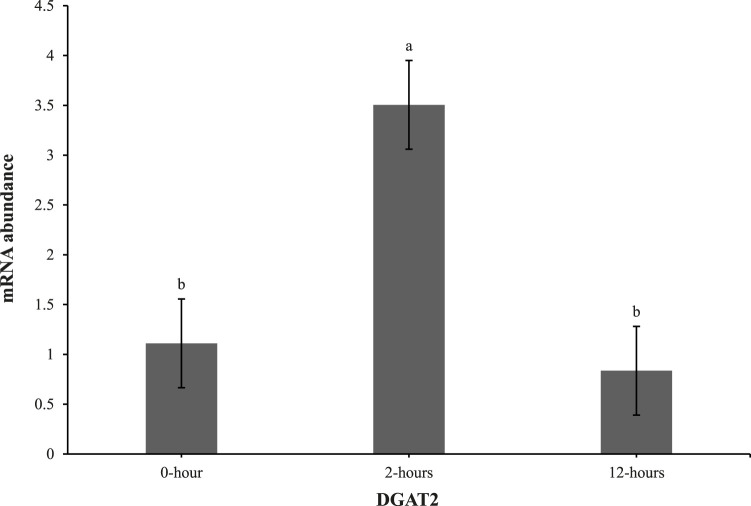
Effect of heat challenge on global DNA methylation (5-methyl cytosine percentage; 5-mC%) in the subcutaneous adipose tissue of control and EHC chicks on day 4 post-hatch. Data (n = 10/timepoint) were analyzed using the Fit Model of jMP and presented as means ± SEM. Different superscripts between timepoints show a difference at *p* < 0.05; Tukey’s test. Chicks hatched at 37.8°C (Control) from day 0 to day 18.5 (E0 to E18.5) or intermittent increase in temperature to 39.5°C and 80% relative humidity from E7 to E16 for 12 h - 07:30–19:30 (EHC). Candling at E18.5 and temperature set to 36.9°C and 50% relative humidity until hatch. Heat challenge = 36°C using three time points: 0 (no heat challenge serving as a control), and 2 and 12 h relative to the start of the challenge.

As shown in Figure 4, the 5-mC% levels in the 0 and 2-h timepoints were comparable. However, at 12 h post heat challenge, the 5-mC% level was elevated compared to the 0 and 2-h time points and was significantly different.

## 4 Discussion

Body weights were not different between the control and EHC chicks at any time point measured. This suggests that EHC did not affect body weight during the heat challenge, which was not surprising given the short timeframe (12 h). These findings are in contrast with those of Zhang et al. (2012) who reported that heat stress significantly affected body and breast weights of Arbor Acres (AA) broilers at 42 days post-hatch subjected to both cyclic (36°C and 23°C for 12-h intervals) and constant heat stress (34°C). The disparity could be due to the sampling time which was day 42 post-hatch compared to 12 h in our experiment. This observation is supported by Mashaly et al. (2004) who observed no difference in body weight of pullets subjected to cyclic (23.9°C for 8 h and 35°C for 4 h and the remaining 12 h as temperature transition) and constant heat stress (35°C), but after week 5 a difference was apparent.

Several studies have employed heat treatment during the incubation of broiler eggs by intermittently increasing the incubation temperature to 39.5°C daily (Collin et al., 2005; Loyau et al., 2013; Moraes et al., 2004; Piestun et al., 2009b). However, excessively high temperatures are undesirable and are likely to result in significantly lower hatchability and livability in both broilers and laying hens (Sgavioli et al., 2016; Yahav et al., 2004a). In broilers, eggshell temperatures above 38.9°C during incubation reduce chick quality, as evidenced by lower yolk-free body mass, shorter chick length, and poor navel conditions (Hulet et al., 2007; Lourens et al., 2005). Furthermore, the organ weights of broilers, especially the heart, are reduced as a consequence of high incubation temperatures (Leksrisompong et al., 2007; Lourens et al., 2007). Liu et al. (2020) reported that heat stress negatively affected feed intake, feed conversion ratio, and consequently body weight gain. Our study agrees with this since NPY, which is considered an appetite stimulant (Newmyer et al., 2013), was reduced at the 0-h timepoint in the EHC group.

To the best of our knowledge, there are no reported data on the effect of embryonic heat stress on adipose tissue mRNA expression in broilers. The analysis of relative mRNA expression levels of key genes involved in adipose tissue metabolism revealed dynamic changes in response to heat challenge at different time points. These findings suggest a complex regulatory mechanism underlying the metabolic response to heat stress in chicks. At 0 h post-heat challenge, the relative mRNA expression levels of C/EBPα, DGAT, PPARγ, and SREBP1 were lower in the EHC group compared to the control, indicating a potential downregulation of adipogenesis-related markers before the start of heat challenge. Speake et al. (1993) reported that more than 90% of the total energy requirements of broilers during embryogenesis is obtained from the yolk. This triggers the development of the subcutaneous fat that becomes visible by E12 which continues to be the main fat depot before the development of the abdominal fat depot that becomes visible by day 7 post-hatch (Kim and Voy, 2021). These could explain the lower expression of adipogenesis-related markers in the EHC at 0 h of the heat challenge. The duration of the EHC protocol used in our experiment coincides with the period of subcutaneous depot development. Higher incubating temperature could have altered the fatty acid oxidation process from the yolk of the EHC chicks during embryogenesis and consequently resulted in less fat deposition after hatch.

Our results demonstrate that EHC significantly impacts the expression of adipogenic genes. Specifically, EHC chicks exhibited an elevated expression of DGAT2 at 2 h post-heat challenge, suggesting a rapid metabolic response to acute thermal stress. DGAT2 is a key enzyme involved in triglyceride synthesis (Chitraju et al., 2019), and its upregulation may indicate an increased capacity for lipid storage and mobilization in response to heat stress. By 12 h post-heat challenge, DGAT2 expression returned to baseline levels, highlighting a transient response likely aimed at restoring homeostasis. Similarly, the expression of PPARγ, a master regulator of adipogenesis (Royan and Navidshad, 2016), was influenced by EHC. While PPARγ levels remained constant at 0 and 2 h post-heat challenge, there was a significant decrease in expression by 12 h. This downregulation suggests that EHC chicks might have a delayed or attenuated adipogenic response to prolonged heat stress, potentially as a protective mechanism to prevent excessive lipid accumulation under thermal stress conditions.

The analysis of global DNA methylation in the subcutaneous adipose tissue revealed 5-mC% levels were similar at 0 and 2 h post-heat challenge. However, at 12 h post-heat challenge, the 5-mC% levels significantly increased. This delayed elevation in DNA methylation suggests an adaptive epigenetic response to prolonged heat stress, potentially impacting gene expression related to stress adaptation and metabolic regulation. Previous reports have demonstrated that environmental stressors can induce DNA methylation changes, affecting stress response and metabolic pathways (Feinberg and Irizarry, 2010; Provençal and Binder, 2015). In our study, the observed increase in 5-mC% levels suggests that these methylation changes may play a role in altering gene expression patterns associated with adipose tissue development and metabolic programming in EHC chicks. Specifically, elevated DNA methylation in key regulatory regions could suppress the expression of genes involved in adipogenesis and lipid metabolism, potentially leading to altered fat deposition and metabolic profiles in these chicks.

Understanding the molecular response to heat stress in adipose tissue is essential for developing strategies to improve heat tolerance and mitigate the negative effects of heat stress on poultry production. Our findings highlight the importance of DNA methylation and other epigenetic modifications in regulating the stress response and metabolic pathways in chicks. Future research should focus on identifying the specific regulatory pathways involved in this process, such as heat shock proteins, inflammatory signaling, and metabolic reprogramming. Additionally, investigating potential interventions, such as nutritional supplements, genetic selection, and management practices, could provide practical solutions to enhance heat tolerance in poultry. By elucidating these mechanisms and developing targeted strategies, we can improve animal welfare and productivity in the poultry industry, particularly in the face of increasing global temperatures.

## Data Availability

The datasets presented in this study can be found in online repositories. The names of the repository/repositories and accession number(s) can be found in the article/Supplementary Material.
